# Genetic regulation and structural changes during tomato fruit development and ripening

**DOI:** 10.3389/fpls.2014.00124

**Published:** 2014-04-23

**Authors:** Paolo Pesaresi, Chiara Mizzotti, Monica Colombo, Simona Masiero

**Affiliations:** ^1^Dipartimento di Bioscienze, Università degli Studi di MilanoMilano, Italy; ^2^Research and Innovation Centre, Fondazione Edmund MachSan Michele all’Adige (Trento), Italy

**Keywords:** tomato, fruit development, ripening, plastid, retrograde and anterograde signaling

## Abstract

Fruits are an important evolutionary acquisition of angiosperms, which afford protection for seeds and ensure their optimal dispersal in the environment. Fruits can be divided into dry or fleshy. Dry fruits are the more ancient and provide for mechanical seed dispersal. In contrast, fleshy fruits develop soft tissues in which flavor compounds and pigments accumulate during the ripening process. These serve to attract animals that eat them and disseminate the indigestible seeds. Fruit maturation is accompanied by several striking cytological modifications. In particular, plastids undergo significant structural alterations, including the dedifferentiation of chloroplasts into chromoplasts. Chloroplast biogenesis, their remodeling in response to environmental constraints and their conversion into alternative plastid types are known to require communication between plastids and the nucleus in order to coordinate the expression of their respective genomes. In this review, we discuss the role of plastid modifications in the context of fruit maturation and ripening, and consider the possible involvement of organelle-nucleus crosstalk via retrograde (plastid to nucleus) and anterograde (nucleus to plastid) signaling in the process.

## FLOWERS ARE THE KEY FACTOR IN THE EVOLUTIONARY SUCCESS OF ANGIOSPERMS

Angiosperms are seed-producing vascular plants, in which the ovules – the precursors of the seeds – develop within the ovary. Developing seeds are enclosed inside the fruits, as also indicated by the term angiosperm, which derives from two Greek words: *angeion*, meaning “vessel” and *sperma*, meaning “seeds.” Estimates of the number of angiosperm species so far described range between 250,000–270,000 and 400,000 (Soltis et al., 2008; Magallón and Castillo, 2009), and they have established themselves in every type of terrestrial and aquatic (fresh and saltwater) habitat. In a letter to J. D. Hooker, written in July 1879 (Darwin and Seward, 1903), Charles Darwin referred to the sudden rise and rapid diversification of angiosperms as “an abominable mystery.” Researchers since have pointed to the innovative aspects of their mode of reproduction – their short reproductive cycles, flower formation, the development of closed carpels and the small size of the male and female gametophytes. The phenomenon of double fertilization, leading to formation of the diploid zygote, and the polyploid endosperm, is also thought to have contributed to the evolutionary success of the angiosperms (Haig and Westoby, 1989; Donoghue and Scheiner, 1992).

A dicot flower can be divided into four concentric but distinct whorls. The sepals of the first or outermost whorl form the calyx, while the corolla, consisting of the petals, lies in the second whorl; in the third whorl is the androecium and the gynoecium develops in the central (fourth) whorl. The female reproductive organ the gynoecium, include the carpels. Carpels are structures that are made up of an ovary and a stigma and contain one or more ovules. One or more carpels are combined into the pistil (ovary, style, stigma), forming the gynoecium as a whole. In the majority of flowering plants, fertilization is required to initiate the transition from ovule to seed, whereas the surrounding carpel(s) and, in some species, other floral organs differentiate into the fruit (Coombe, 1975). Furthermore, fruits represent a major evolutionary innovation, are essential for plant reproduction and adaptation, and greatly enhance the efficiency of seed dispersal. The ability to germinate and grow far away from the parent plant allows angiosperms to colonize new areas, reducing the risk of inbreeding and sibling competition (Willson and Traveset, 2000).

## CLASSIFICATION OF ANGIOSPERM FRUITS

According to Brooks, fruits are “matured carpels with or without accessory structures and/or seeds” (Coombe, 1976). Nitsch, on the other hand, defined them as “the tissues which support the ovules and whose development is dependent upon the events occurring in these ovules.” Nitsch’s definition thus includes the “false” fruits, so called because extracarpellary tissues give rise to much of the fleshy tissue that bears or encloses the true fruits. Examples include pomes and strawberries, which form by the expansion and proliferation of the receptacle (Perkins Veazie, 1995; Velasco et al., 2010).

Based on their texture, fruits are mainly divided into two major groups: fleshy and dry. At maturity, dry fruits are characterized by dry pericarp (Simpson, 2011) and they can be further classified into dehiscent or indehiscent fruits. Dehiscent fruits open and release the mature seeds, while the indehiscent fruits do not disperse the seeds. It has been proposed that dry dehiscent fruits, found in all major clades of angiosperms, correspond to the ancestral type (Knapp, 2002), whereas the *Arabidopsis* silique with its specialized dehiscence zone may be a more recent evolutionary invention (Mühlhausen et al., 2013).

With regard to fleshy fruits Darwin writes “this (a fruit’s) beauty serves merely as a guide to birds and beasts in order that the fruit may be devoured and the manured seeds disseminated.” Darwin recognized that seeds protected by a fleshy fruit become more attractive for animals, which in turn play an essential role in their dispersal. The attractiveness and juiciness of fleshy fruits originate in the important cytological modifications which the parenchymal tissue undergoes during ripening – including chlorophyll degradation, accumulation of carotenoids and flavonoids, development of an aroma and flavors, and softening of the pulp (Willson and Whelan, 1990; Rodríguez et al., 2013).

Nevertheless, the specific biochemical programs that result in ripening phenomena vary among species, as highlighted by the fact that fleshy fruits can be further divided into two categories: climacteric and non-climacteric. The term “climacteric” was initially proposed to emphasize the dramatic increase in fruit respiration – marked by a burst of CO_2_ production (Biale, 1964). However, climacteric fruit ripening is actually stimulated by ethylene (Razali et al., 2013), although ethylene-dependent and -independent genes have been identified both in climacteric and non-climacteric fruits (Barry and Giovannoni, 2007). Intriguingly, recent data indicates that also the dry *Arabidopsis* silique shows a climacteric behavior as suggested by the patterns of ethylene production and respiration, and by its response to ethylene exogenous application (Kou et al., 2012).

## DEVELOPMENT AND MATURATION OF THE TOMATO FRUIT

Among climacteric fleshy fruits, the tomato proved attractive to early inhabitants of the Americas, who initiated its domestication by selecting varieties with fruits larger than those of the wild ancestor *Lycopersicon esculentum* cv.* cerasiforme* (Tanksley, 2004; Peralta et al., 2006; Cong et al., 2008) – a process which has gone on up to the present day, as shown by the large collection of cultivars now in use, characterized by fruits with different sizes and shapes (Tanksley et al., 1996; Grandillo et al., 1999; Tanksley, 2004; Bai and Lindhout, 2007). Moreover, tomato fruits contribute more nutrients to the diet than any other fruit or vegetable, since they contain relatively large amounts of lycopene (Chalabi et al., 2004), vitamins C and A, potassium, folic acid and many other metabolites. Lycopene, for instance, has a strong antioxidant capacity because of its great ability to trap peroxyl radicals. Epidemiological studies recommend the consumption of foods containing high concentrations of lycopene, since it reduces the risk for certain types of cancer, including prostate cancer (Gann et al., 1999; Giovannucci et al., 2002; Jian et al., 2005).

**FIGURE 1:**
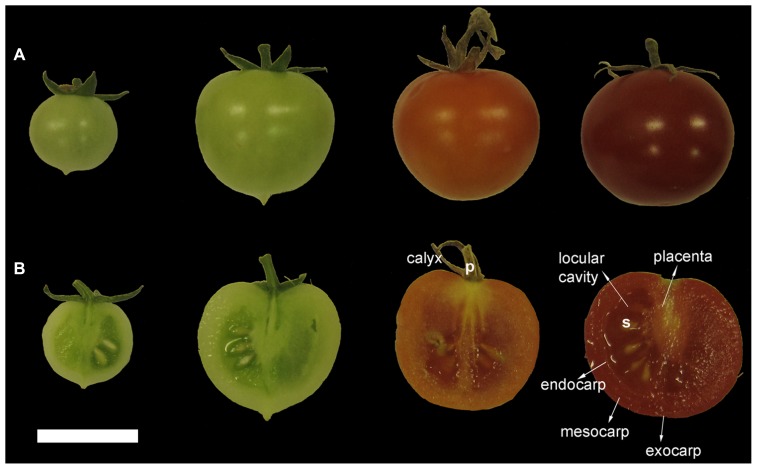
**Different stages of tomato fruit development and anatomical details. (A)** Tomato fruit development can be divided into different stages: IG, immature green; MG, mature green; BR, orange-breaker; and RR, red ripening stages are shown. **(B)** Transverse sections of fruits corresponding to the developmental stages shown in **(A)**. p, pedicel; s, seed. Scale bar: 2 cm.

From a botanical point of view, the tomato fruit is a berry, which can be bi- or multilocular (**Figure 1**). The septa of the carpels divide the ovary and the fruit into two or more locules. Seeds develop attached to the placenta, a parenchymatous tissue, which becomes gelatinous and fills the locular cavities during fruit development and maturation (Grierson and Kader, 1986; Ho and Hewitt, 1986; Bertin, 2005; Mintz-Oron et al., 2008).

After fertilization, the ovary wall is transformed into the pericarp, which can be divided into three different structures: exocarp, mesocarp, and endocarp. The external exocarp consists of a cuticle layer that thickens as the fruit ages, and the skin, which includes an epidermal cell layer and three to four layers of a collenchymatous tissue, in which starch accumulates and few plastids are retained (Esau, 1953; Varga and Bruinsma, 1986; Joubès et al., 2000; Lemaire-Chamley et al., 2005; Mintz-Oron et al., 2008). The mesocarp, the intermediate layer, is a parenchymatous tissue formed by big cells with large vacuoles (Joubès et al., 2000; Lemaire-Chamley et al., 2005; Mintz-Oron et al., 2008). The cells of the mesocarp commonly undergo six to eight rounds of DNA duplication (endocycles) reaching ploidy levels of up to 512C (Bourdon et al., 2010) and are reminiscent of the palisade cells of leaves (Gillaspy et al., 1993) since they contain several chloroplasts, the organelle where photosynthesis occurs and produces up to 20% of fruit photosynthate, whereas the rest of photoassimilates are imported from source leaves (Hetherington et al., 1998). Nevertheless, the role of fruit photosynthesis in fruit metabolism and development is not fully understood. Early shading experiments (Tanaka et al., 1974) as well as the fruit-specific antisense inhibition of the chloroplastic Fructose 1,6-bisphosphatase (FBPase) indicated an important contribution of fruit photosynthesis to fruit yield, as shown by the reduction in weights of ripe fruits with reduced photosynthetic performance (Obiadalla-Ali et al., 2004). On the contrary, tomato lines exhibiting a fruit-specific reduction in the expression of glutamate 1-semialdehyde aminotransferase (GSA) and, as a consequence, lowered chlorophyll levels and photosynthetic activity, displayed almost no differences in fruit size and weight (Lytovchenko et al., 2011). However, these lines were characterized by a striking reduction in the rate of seed set as well as an altered seed morphology, which displayed a much reduced embryo-to-seed ratio, indicating that fruit photosynthesis is an important source of carbone assimilate for proper seed set and establishment under normal growth conditions.

Finally, the endocarp, the innermost structure, consists of a single cell layer adjacent to the locular region (Mintz-Oron et al., 2008; Xiao et al., 2009; de Jong et al., 2010).

Fertilization (stage 0) normally initiates the development of the tomato fruit, which proceeds through several major stages (Picken, 1984; Gillaspy et al., 1993; **Figure 1**). The first stage, immediately after fertilization, is characterized by rapid cell division, leading to a progressive increase in pericarp cell number. The end of this stage – around two weeks after pollination – is marked by a sharp fall in the rate of cell division, when the fruit is about 0.8–1 cm in diameter. During the second stage, fruit growth relies on cell expansion and leads to a significant increase in weight. Cell expansion coincides with endoreduplication (Bergervoet et al., 1996). By the end of this stage fruits have a diameter of around 2 cm. During the third phase, the fruit enters the mature green (MG) stage (Ho and Hewitt, 1986; Giovannoni, 2004; Czerednik et al., 2012) and attains its final size, which varies greatly among cultivars and is very susceptible to environmental influences (Chevalier, 2007).

Roughly 2 days after reaching the MG stage, the tomato fruit undergoes an extensive metabolic reorganization, which marks the beginning of the fruit ripening process (Ho and Hewitt, 1986). Two main phases can be distinguished, which are referred to as the breaking (BR) and the ripening (RR) stages. Conversion of chloroplasts into chromoplasts signals the BR phase, as indicated by the change in color to yellow-orange, owing to carotenoid accumulation, and concomitant chlorophyll degradation (**Figure 1**). Interestingly, proper ripening in tomato occurs only if fruits are harvested after having completed at least 40% of their normal growth: even exogenous application of ethylene fails to induce ripening in undeveloped locules.

At the end of the ripening process the abscission zone (AZ) is formed in the pedicel (Szymkowiak and Irish, 1999; Mao et al., 2000) to allow fruit to fall once mature. AZs differentiate at predetermined positions and contain a group of small cells lacking large vacuoles; in tomato, differentiation of the pedicel AZ is controlled by the MADS-box transcription factor JOINTLESS (Szymkowiak and Irish, 1999; Mao et al., 2000).

## TOMATO: A MODEL SPECIES FOR FLESHY FRUIT STUDIES

Tomato is an ideal model plant for studying climacteric fruit ripening. Several tomato gene banks have been established and more than 75,000 accessions of tomato are maintained (Larry and Joanne, 2007; Minoia et al., 2010; Okabe et al., 2011; Saito et al., 2011). In addition, several mutants affected in fruit size, shape, development, and ripening have been isolated (Liu et al., 2002; Tanksley, 2004; Xiao et al., 2009; Rodríguez et al., 2011). Recently, the genome of *Solanum lycopersicon* cv. “Heinz 1706” (Tomato Genome Consortium, 2012; Aoki et al., 2013) has been fully sequenced and made publicly available. The predicted size of its diploid genome is approximately 900 megabases (Mb), distributed on 12 chromosomes, more than 75% of which is heterochromatin and largely devoid of genes. Around 33,000 genes have been predicted and some 5000 genes are preferentially expressed in fruits (Tikunov et al., 2013). With its short generation time, and the availability of a routine transformation technology, mapping populations, and microarrays of mapped DNA markers, tomato is a highly tractable experimental system. Several “omics” tools (transcriptomics, proteomics, and metabolomics) have been used to explore fruit formation and development (Alba et al., 2004; Fei et al., 2004; Fernie et al., 2004; Rose et al., 2004; Alba et al., 2005; Moore et al., 2005), leading to the genetic characterization of several important traits that have been selected during tomato domestication.

For instance several loci, named *FRUIT WEIGHT* (*FW*), have been recognized as key regulators of fruit mass (Grandillo et al., 1999; Paran and van der Knaap, 2007). Thus the *FW2.2* allele increases *FW* by up to 30% and is found in commercial cultivars (Frary et al., 2000), whilst the small-fruited allele is present in wild tomato species. *FW2.2* encodes a plasma membrane-localized protein that inhibits cell division; therefore low levels of *FW2.2* mRNA promote cell cycling, leading to bigger fruits containing more and larger cells (Nesbitt and Tanksley, 2001).

Tomato fruit size is also influenced by locule number. Two loci, *fasciated* (*f* or *fas*) and *locule number* (*lc*), affect floral meristem size and organ/carpel number. *FAS* encodes a YABBY transcription factor, and it is down-regulated in the high-locule-number mutant (Barrero and Tanksley, 2004). The *lc* locus seems to correspond to two single nucleotide polymorphisms (SNPs) that map close to the tomato homolog of the *WUSCHEL* gene in *Arabidopsis thaliana*; however, no deregulation of *WUS* has been observed in low- or high-locule cultivars (Muños et al., 2011). In *Arabidopsis* the WUS protein is involved in stem cell maintenance, and its up-regulation leads to the formation of extra carpels (Carles et al., 2004).

## GENETIC AND HORMONAL REGULATION OF FRUIT DEVELOPMENT: A TOMATO PERSPECTIVE

### THE GENETICS OF FRUIT FORMATION

Fruit formation requires intimate exchange of developmental information between ovules and carpels. Signals that stimulate fruit development may be produced by pollen grains (O’Neill, 1997; O’Neill and Nadeau, 1997) and in ovules once fertilization has successfully occurred (Gillaspy et al., 1993), leading to alteration of the developmental fate of pistils from senescence to fruit set (Vercher et al., 1984; van Doorn and Woltering, 2008).

Since fruits are mature gynoecia, carpel patterning anticipates fruit architecture. Carpel identity is in turn controlled by the homeotic genes of class C, which includes all the members of the AGAMOUS sub-clade (AG; Dreni et al., 2011), named for the first member identified, in *A. thaliana* (Yanofsky et al., 1990; Becker and Theiβen, 2003). Several comparative studies indicate that the functions of members of the AG sub-clade are conserved from monocots to basal core eudicots (Bowman et al., 1989; Bradley et al., 1993; Pnueli et al., 1994; Mena et al., 1996; Davies et al., 1999; Pan et al., 2010; Yellina et al., 2010; Dreni et al., 2011).

**FIGURE 2:**
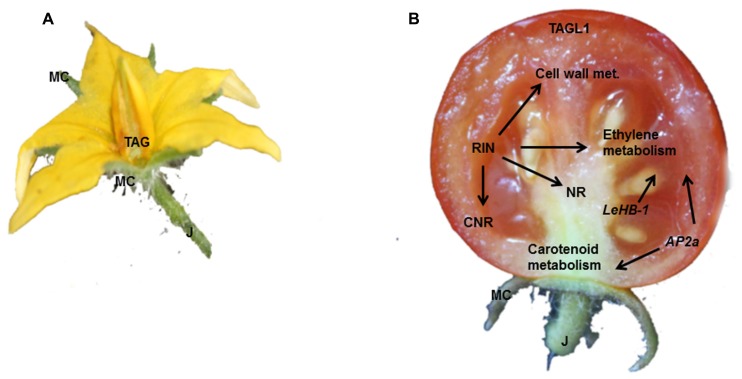
**The key regulators involved in fruit commitment, formation and ripening. (A)** The MADS-box transcription factors TAG controls pistil identity, whist J regulates the pedicel abscission zone differentiation. **(B)** TAGL1, RIN, AP2a, and LeHB-1 all impact the ethylene metabolism; although RIN controls also the transcription of genes involved in cell wall modification.

In tomato, as in snapdragon (Mizzotti et al., 2014), there are two *AG*-like genes (**Figure 2**), *TAGL1* and *TAG (TOMATO AG-LIKE 1* and* TOMATO AG*). Silencing of *TAGL1* influences fruit ripening, without affecting floral organ specification (Vrebalov et al., 2009; Giménez et al., 2010; Pan et al., 2010). In particular, *tagl1* fruits are characterized by a thinner pericarp, reduced firmness at the BR stage, and the maintenance of plastids in the collenchyma cells of the pericarp; consequently *tagl1* fruits accumulate more chlorophyll and lutein than wild-type fruits (Itkin et al., 2009; Vrebalov et al., 2009; **Table 1**).

**Table 1:** Mutations associated with defects in fruit maturation and ripening.

Tomato fruit mutants			
Mutant	Phenotype/tissues affected	Reference	Gene product
*tag*	Flower meristem and inner whorl fate determination	Pnueli et al. (1994), Pan et al. (2010)	MADS-box transcription factor
*tagl1*	Chlorophyll and carotenoid accumulation	Vrebalov et al. (2009), Giménez et al. (2010), Pan et al. (2010)	MADS-box transcription factor
	Plastids present in the collenchyma of the exocarp
*rin*	Ripening delay	Vrebalov et al. (2002)	MADS-box transcription factor
*mc*	Sepal development	Vrebalov et al. (2009)	MADS-box transcription factor
*j*	Abscission zone formation	Szymkowiak and Irish (1999), Mao et al. (2000)	MADS-box transcription factor
*Nr*	Ripening delay	Lanahan et al. (1994), Yen et al. (1995)	Ethylene receptor
*CNR*	Ripening delay	Manning et al. (2006)	SBP transcription factor
*AP2a*	Regulation of carotenoid and chlorophyll metabolism	Chung et al. (2010), Karlova et al. (2011)	AP2 transcription factor
*slarf7* (RNAi)	Parthenocarpy	de Jong et al. (2009)	Auxin-responsive factor 7
*gr*	Ripening delay	Barry and Giovannoni (2006)	RTE-like proteins

Recently, the semi-dominant insertion mutation *Arlequin (Alq*) has been mapped and found to correspond to an altered form of the *TAGL1* gene (Giménez et al., 2010). In *Alq* plants, sepals are transformed into fruits which undergo a ripening process, like the true fruits originated by the pistils. Thus this mutant phenocopies transgenic lines that overexpress *TAGL1* (Itkin et al., 2009; Vrebalov et al., 2009; Giménez et al., 2010).

*TAG1*, on the other hand, has been shown to be necessary for determination of stamens and carpels, as revealed by the effects of its down-regulation using antisense and RNAi approaches (Pnueli et al., 1994; Pan et al., 2010). Indeed, pistils are replaced by a reiteration of flowers in transgenic plants expressing *TAG1* antisense (Pnueli et al., 1994) – just as in the *Arabidopsis ag* mutant (Yanofsky et al., 1990). In contrast, virtually complete silencing of *TAG1* by RNAi does not affect pistil fate in this way instead, pistils develop into red fruits, indicating a loss of determinacy (Pan et al., 2010).

Besides *AG* genes, several other MADS-box transcription factors are involved in fruit formation and maturation (**Figure 2**). Vrebalov et al. (2002) showed that the classical mutation *rin* disrupts the function of RIN-MADS. RIN-MADS lies very close to another MADS-box gene, *MACROCALYX* (*MC*), which is also silenced in *rin* plants. However, antisense repression of *RIN-MADS* and *MC* confirmed that only RIN-MADS is necessary for tomato ripening (**Table 1**).

Several independent groups have described a plethora of direct targets for RIN-MADS (Ito et al., 2008; Fujisawa et al., 2011, 2013; Martel et al., 2011). Thus RIN-MADS binds to regulatory regions of several genes, whose products are involved in fruit metabolism and ripening, and transcriptionally regulates enzymes involved in cell wall (*Polygalacturonase, PG*;* β-Galactosidase 4, TBG4*; *Endo-*(*1,4*)*-β-mannanase 4, LeMAN4*; α*-Expansin 1, LeEXP1*), and carotenoid metabolism. RIN-MADS is also a master regulator of ethylene biosynthesis in developing fruits, acting via the control regions of the genes *LeACS2* (*1-aminocyclopropane-1-carboxylic acid synthase 2*)*, LeACS4*, *LeACO1* (*ACC oxidase 1*). Moreover, RIN-MADS stimulates the transcription of *Lipoxygenase* (*Lox*), the product of which catalyzes the dioxygenation of 1,4 pentadiene *cis*-polyunsaturated fatty acids to their hydroperoxide derivatives (HPO), resulting in the production of volatile compounds that contribute to fruit flavor and aroma (Yilmaz, 2001).

RIN-MADS also binds and activates the promoter of *NEVER RIPE* (*NR*; Lanahan et al., 1994; Yen et al., 1995), which encodes an ethylene receptor protein. The loss of this protein/DNA interaction explains the delay in ripening seen in *rin* mutants (Klee and Tieman, 2002).

RIN-MADS also positively stimulates the transcription of *colorless non-ripening* (*CNR*), which codes for a SQUAMOSA-PROMOTER BINDING PROTEIN (Cardon et al., 1999; Manning et al., 2006) whose absence causes delay in fruit ripening and softening as a consequence of reduced ethylene production (Thompson et al., 1999). The interaction between *CNR* and RIN-MADS has been shown to be regulated by a complex mechanism. The *CNR* promoter is progressively demethylated during ripening, but in *cnr* mutants the promoter remains hypermethylated, which prevents RIN-MADS from binding to it (Zhong et al., 2013). Zhong et al. (2013) observed that the methylation states of several RIN-MADS targets change during ripening, indicating that progressive demethylation is necessary for RIN-MADS binding. Indeed, these authors showed that tens of thousands of sites in the tomato epigenome undergo modification during fruit development.

Transcriptomic studies suggest that many more transcription factors are involved in the regulation of ripening (Vriezen et al., 2008; Pascual et al., 2009) and recently members of the APETALA2 family have been shown to play a role in the process. For instance, the tomato *APETALA2a* gene (Karlova et al., 2011) participates in the control of fruit ripening by regulating genes involved in ethylene and auxin signaling, and in the differentiation of chloroplasts into chromoplasts. Down-regulation of *AP2a* in transgenic fruits is associated with the accumulation of β-carotene at the expense of lycopene (Chung et al., 2010). Ethylene metabolism is also controlled by the transcription factor *Lycopersicum esculentum Homeobox-1* (*LeHB-1*), which binds to control regions of *ACO1* (Lin et al., 2008).

### HORMONES AND FRUIT DEVELOPMENT

Fruit development and maturation is tightly controlled by hormone homeostasis (Pandolfini, 2009). Indeed, several findings indicate that manipulation of hormone homeostasis is able to induce fruit development and ripening in the absence of fertilization – a phenomenon known as parthenocarpy.

Thus treatment of unpollinated flowers with auxins is sufficient to stimulate fruit growth in tomato and other horticultural plants, indicating that administration of the hormone can substitute for the signals provided by pollination and fertilization (Nitsch, 1952). Auxin homeostasis can be altered by manipulating its synthesis, perception or signaling. For example, AtARF8 (*Arabidopsis thaliana* auxin response factor 8) and tomato ARF7 have both been implicated in fruit initiation. *atarf8* mutants develop parthenocarpic fruits, while tomato fruits that express the *arf8-4* allele are seedless (Wang et al., 2005). Parthenocarpy can also be induced by silencing *ARF7* in tomato via RNA interference (de Jong et al., 2009).

Besides auxins, gibberellins (GA) play an important role in coordinating fruit growth and seed development. Active GA induce fruit set in crop plants and in *Arabidopsis* (Gillaspy et al., 1993; Dorcey et al., 2009), in agreement with transcriptomic analyses showing that GA biosynthesis genes are highly expressed in pollinated ovaries (Lemaire-Chamley et al., 2005). Inhibition of GA production by paclobutrazol has negative effects on fruit growth and seed set in tomato (Serrani et al., 2007), while the transgenic tomato lines *pat2* and *pat3/4* show overexpression of GA biosynthetic genes in their parthenocarpic fruits (Rotino et al., 2005). This is consistent with the finding that silencing of *DELLA* genes (Hauvermale et al., 2012), which code for negative regulators of GA signaling, results in the development of parthenocarpic fruit in both tomato and *Arabidopsis* (Martì et al., 2007; Dorcey et al., 2009; Fuentes et al., 2012).

Tomato is a climacteric fruit and its ripening is dependent on an ethylene burst. Conversely, in several tomato mutants in which ripening is delayed (including *rin, cnr* and *nr*), ethylene production is compromised. Synthesis of ethylene depends on the action of two enzymes, ACC synthase (ACS) and ACC oxidase (ACO). ACS converts S-adenosylmethionine into 1-aminocyclopropane-1-carboxylate, which is subsequently transformed into ethylene by ACO. ACC synthases in tomato are encoded by a multigene family (Zarembinski and Theologis, 1994; Oetiker et al., 1997), but only *LeACS2* and *LeACS4* are up-regulated during climacteric fruit ripening (Olson et al., 1991; Barry et al., 1996, 2008; Baldwin et al., 2000; Alba et al., 2005; Barry and Giovannoni, 2006), and the down-regulation of *LeACS2* and *ACO* delays ripening and the transgenic tomato fruits increase their shelf life (Xie et al., 2006).

*NEVER RIPE* is a semi-dominant mutation that affects one of the seven ethylene receptors (*Lycopersicum esculentum ethylene receptor*, *LeETR1-7*) present in the tomato genome. Of these seven genes, however, only* LeETR4, LeETR6* and* Nr* (*LeETR3*) are strongly expressed during fruit ripening.

*Green-ripe* (*Gr*) is also a dominant non-ripening mutant (Barry and Giovannoni, 2006; Xie et al., 2013), whose phenotype is due to misexpression of the *Gr* gene in developing fruits and organs, where it is normally not active. GR codes for a homolog of the *Arabidopsis* RTE1 protein (Barry and Giovannoni, 2006), a factor that is able to bind to and modify ethylene receptors, although how it affects receptor function remains unclear.

## THE CHLOROPLAST TO CHROMOPLAST TRANSITION AND NUCLEUS-PLASTID COMMUNICATION

The onset of fruit ripening and the consequent reprogramming of cellular metabolism is most strikingly reflected in the conversion of fully developed chloroplasts into chromoplasts, a type of plastid that accumulates massive amounts of colorful carotenoids to attract insects and mammals that facilitate the dispersal of the seeds contained in fleshy fruits (Egea et al., 2010).

The chloroplast to chromoplast transition involves various structural modifications, including changes in the density and size of the organelle (Rosso, 1968; Spurr and Harris, 1968; Harris and Spurr, 1969), breakdown of chlorophylls, disruption of the thylakoid membrane and the aggregation of carotenoids into crystals (Egea et al., 2011). Scanning confocal microscopy analyses indicate that at the MG stage of tomato development only chloroplasts are present, mainly located in the mesocarp cells.

During the breaker stage (BR), plastids begin to accumulate carotenoids, with the rate of accumulation of lycopene being three- to fourfold higher than that of chlorophyll decline (Trudel and Ozbun, 1970; Wu and Kubota, 2008; Egea et al., 2011).

From a structural point of view, the dedifferentiation of chloroplasts into chromoplasts begins with the breakdown of starch granules and the lysis of thylakoid membranes (Ljubesić et al., 1991; Egea et al., 2010). Concomitantly, new membranes are formed, which are derived from the plastid inner envelope and become sites of carotenoid accumulation and crystal formation (Simkin et al., 2007). During the transition plastoglobules and stromules increase in size and number (Harris and Spurr, 1969; Gray et al., 2001; Kwok and Hanson, 2004; Egea et al., 2010). Plastoglobules serve to sequester lipids and carotenoids (Klee and Giovannoni, 2011; Nogueira et al., 2013), whereas the stromules provide extra surface area for the import of novel plastid proteins (Kwok and Hanson, 2004).

The situation just described is typical for immature chromoplasts at early stages of differentiation. At the full ripening stage, the plastids in fruits are almost exclusively chromoplasts. Interestingly, using real-time recording of the transition occurring in the mesocarp tissues, Egea et al. (2011) were able to demonstrate that the transition from chloroplasts to chromoplasts occurs more synchronously within individual cells than between different cells of the fruit tissue. Moreover, since these authors found no evidence for *de novo* formation of plastids, they concluded that all chromoplasts originate from pre-existing chloroplasts, as previously suggested (Pyke and Howells, 2002; Waters et al., 2004; Egea et al., 2011).

Over 95% of the ~3000 proteins found in the chloroplast are encoded in the nuclear genome, translated in the cytoplasm and then imported into the organelle (Richly and Leister, 2004; Li and Chiu, 2010). Therefore, the transition from chloroplast to chromoplast must involve extensive exchange of information between the nucleus and the plastids, in order to regulate the plastid proteome and ensure that the organelle can meet the changing metabolic and energy demands of the cell (Chi et al., 2013). This notion is supported, for example, by the fact that mutation of the tomato *lutescent2* locus (*l2*), encoding a chloroplast-targeted zinc metalloprotease, delays the onset of fruit ripening, which implies the existence of a chloroplast-derived signal that stimulates ripening (Barry et al., 2012).

Communication between plastids and the nucleus, and the nature of plastid-derived signals, have been widely studied in model organisms such as *A. thaliana*, and this has led to the identification of several key factors that are essential for chloroplast biogenesis (biogenic control) and adaptation to physiological and environmental conditions (operational control; for a review see Woodson and Chory, 2008; Chi et al., 2013).

Interestingly, the expression of these factors is maintained in *Arabidopsis* and tomato fruits at different developmental stages, suggesting a possible involvement of anterograde (nucleus-to-plastid) and retrograde (plastid-to-nucleus) signaling pathways in fruit maturation and ripening.

### THE ANTEROGRADE PATHWAY

During anterograde regulation, nucleus-encoded transcriptional and post-transcriptional regulators convey information about cell type to the plastid, and nuclear genes direct the synthesis and delivery of proteins that are appropriate for the organelle’s development, division and differentiation into chloroplasts, amyloplasts, chromoplasts, and other plastid types (Leon et al., 1998; Raynaud et al., 2007). In general, nucleus-encoded post-transcriptional regulators, such as proteins of the tetratrico-peptide-repeat (TPR) and pentatrico-peptide-repeat (PPR) families (D’Andrea and Regan, 2003; Nakajima et al., 2012), bind to specific chloroplast mRNAs, and control their maturation and/or stabilization by acting as adaptors for enzymes of chloroplast RNA metabolism. Alternatively, they regulate protein synthesis initiation and/or elongation by recruiting the chloroplast translation machinery to specific mRNAs (Blatch and Lässle, 1999; Shikanai and Fujii, 2013). Through these processes, TPR, PPR, and other types of imported proteins mediate subtle regulatory changes, such as the assembly and abundance of specific protein complexes in response to developmental and environmental stimuli.

Conversely, large-scale developmental switches, such as the reprogramming that takes place during the chloroplast-to-chromoplast transition (Leon et al., 1998), lead to a general increase in transcription and in differential transcript accumulation. The plastome of higher plants is transcribed by two quite different transcription systems that originate from a cyanobacterial- and proteobacterial-like endosymbiont respectively (Maliga, 1998; Liere et al., 2011). The cyanobacterial ancestor of chloroplasts provided a eubacterial-type RNA polymerase (PEP) whose four-subunit core, comprising α, β, β′, and β^′ ′^ proteins, is encoded by the plastid genes *rpoA*, *rpoB*, *rpoC1,* and *rpoC2*. The PEP plays a prominent role in the expression of photosynthesis-related genes in leaf chloroplasts, but it is also present in dry seeds and is active during germination. The activity and specificity of PEP is regulated by nucleus-encoded sigma-like transcription factors (SIGs). In *Arabidopsis* six such sigma factors (SIG1-6) have been identified, and they appear to have distinct roles during embryonic photosynthesis (SIG5), seed maturation and germination (SIG3) and very early plant development (SIG2 and SIG6).

Two nuclear genes encode the plastid proteobacterial-like RNA polymerases (NEPs), named RPOTp and RPOTmp, the latter being targeted to and active in mitochondria also (Liere et al., 2011). NEPs are active in the expression of housekeeping genes in plastids, and they play an important role in the build-up of the plastid transcriptional and translational apparatus during stratification, germination and early seedling development.

Putative homologs of *Arabidopsis* anterograde signaling factors can be identified in tomato, using BLAST queries of transcript (cDNA ITAG release 2.31) and protein databases (ITAG release 2.31) available on the SGN website^1^. In addition, the expression patterns of the corresponding mRNAs in leaves and in tomato fruits at different times during maturation (1-cm fruit, 2-cm fruit and MG fruit, BR, and RR stages) can be assessed with the aid of the Tomato eFP browser^2^ (**Table 2**).

**Table 2:**
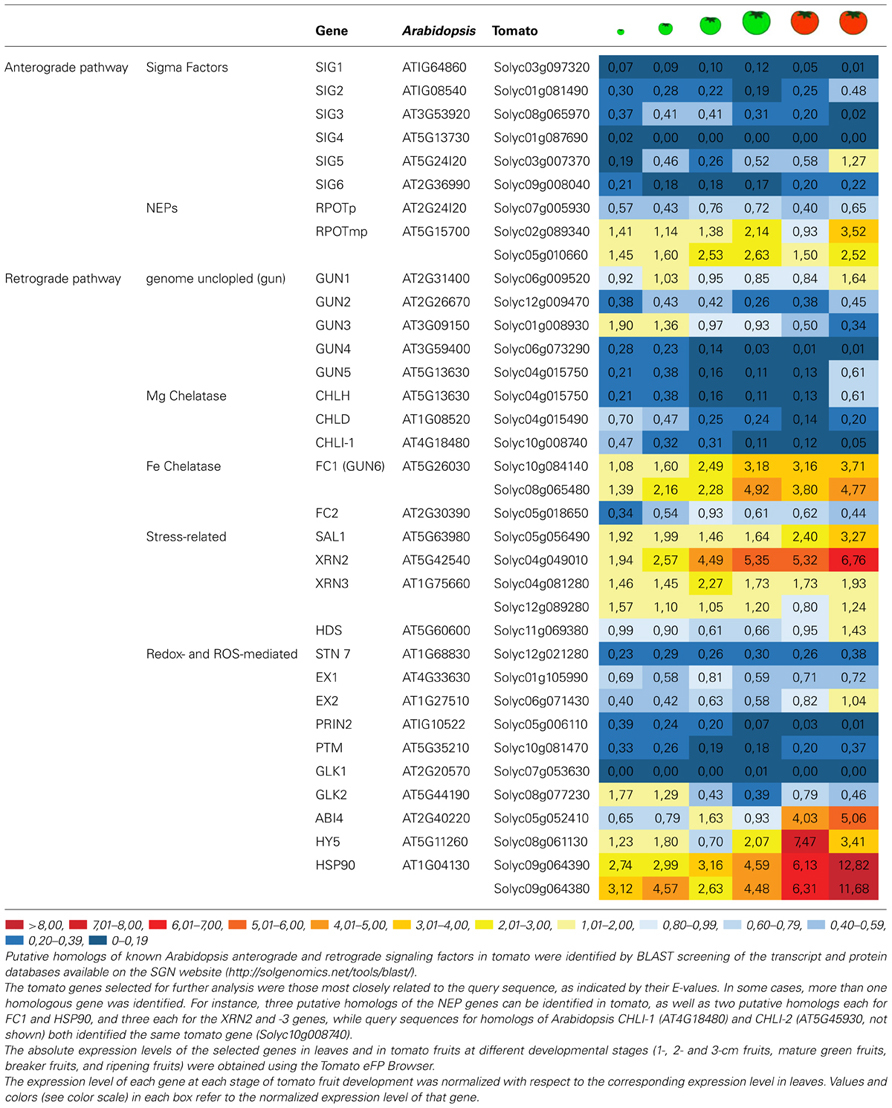
Relative expression levels of the putative homologs of *Arabidopsis* anterograde and retrograde factors in developing tomato fruit.

As expected, all putative homologs of sigma factors appear to be down-regulated in fruit with respect to leaves, confirming their predominant role in the PEP-mediated expression of photosynthesis-related genes. The only exception is represented by the *SIG5* homolog (*Solyc03g007370*), which is expressed at slightly higher levels in ripening tomato fruits than in leaves.

Conversely, the three putative tomato homologs of plastid proteobacterial-like RNA polymerases (Solyc07g005930; Solyc02g089340; Solyc05g010660) display intriguing expression patterns in developing fruits. The closest homolog of RPOTp (*Solyc07g005930*) is down-regulated in fruit, while the other two (*Solyc02g089340; Solyc05g010660*), which are more similar to the RPTOPmp form found in both mitochondria and plastids, show higher expression levels relative to leaves in all the different fruit stages. In particular, their expression levels follow very similar patterns, with a first peak occurring at the MG fruit stage and a second at the ripening stage. These observations imply a very strict coordination of mitochondrial and plastid transcription activities during fruit formation and maturation.

### THE RETROGRADE PATHWAY

The term retrograde signaling refers to the regulation of nuclear gene expression in response to the developmental stage and functional state of the plastids, including plastid differentiation (Enami et al., 2011). In the classical scenario, the retrograde signal is generated in the plastids, then exported, and traverses the cytosol to act in the nucleus. Several metabolites have been proposed to act as messengers during retrograde signaling. These include (1) tetrapyrroles (Mg-protoporphyrin IX or heme; Strand et al., 2003; Woodson et al., 2011); (2) 3-phosphoadenosine-5-phosphate (PAP; Estavillo et al., 2011); and (3) methylerythritolcyclodiphosphate (MEcPP; Xiao et al., 2012).

The involvement of tetrapyrroles in retrograde signaling in *Arabidopsis* was revealed by the identification of *g*enome *un*coupled (*gun*) mutants that, unlike wild type, continue to express photosynthesis-related nuclear genes including *ribulose bisphosphate carboxylase small subunit* (*RBCS*) and *light harvesting complex of photosystem II* (*Lhcb*s) even when chloroplasts have been photobleached by exposure to the herbicide norflurazon (Susek et al., 1993). In particular, GUN2, GUN3, and GUN6 are involved in the iron branch of tetrapyrrole biosynthesis leading to heme and phytochromobilin, and code for the enzymes heme oxygenase 1, phytochromobilin synthase, and Fe-chelatase 1, respectively. GUN4 and GUN5, on the other hand, operate in the magnesium branch that leads to chlorophylls, and form part of the Mg-chelatase enzymes together with CHLH, CHLD, CHLI-1, and CHLI-2 subunits (for a review, see Chi et al., 2013). However, the role of Mg-protoporphyrin IX (Mg-ProtoIX) as a plastid signal has been questioned, since its accumulation following norfluorazon treatment has not been observed in two independent studies (Mochizuki et al., 2008; Moulin et al., 2008). Consequently, it was suggested that either rapid changes in the flux through the tetrapyrrole pathway, or the accumulation of Mg-ProtoIX in a specific cellular compartment could be the origin of the plastid signal (Mochizuki et al., 2008; Moulin et al., 2008); however, these aspects deserve further investigations.

A novel role as a retrograde signaling messenger was recently assigned to PAP (Estavillo et al., 2011). PAP accumulates in the chloroplast under drought conditions or upon exposure to excess light, and functions as a mobile signal that alters nuclear RNA metabolism by inhibiting exoribonucleases (XRNs). Evidence for PAP-mediated chloroplast-to-nucleus communication came with the identification of the *alx8* mutant, which exhibits constitutive up-regulation of genes normally induced by high-light stress. The *alx8* phenotype is caused by a lesion in SAL1, a phosphatase that regulates PAP levels by dephosphorylating PAP to adenosine monophosphate (AMP).

Recently, a role as a retrograde signaling metabolite has been also reported for MEcPP, a precursor of isoprenoids produced by the plastidial methylerythritol phosphate (MEP) pathway (Xiao et al., 2012). This finding came from the observation that *Arabidopsis* plants showing constitutive expression of selected stress-responsive nuclear genes also accumulated high levels of MEcPP, as a consequence of a lesion in the enzyme HDS, which is responsible for the conversion of MEcPP to HMBPP in the plastid-specific, non-mevalonate MEP pathway.

Changes in chloroplast homeostasis are also closely associated with changes in the redox state of the thylakoid electron transport chain (Baier and Dietz, 2005), particularly the redox state of the plastoquinone pool (PQ) and increases in the levels of reactive oxygen species (ROS), which also trigger retrograde signaling processes (Apel and Hirt, 2004; Asada, 2006). Components of the redox and ROS signaling circuits have been identified by genetic analysis in *A. thaliana*. They include STN7, a dual-function thylakoid protein kinase required for state transitions and photosynthetic acclimation (Bonardi et al., 2005; Pesaresi et al., 2009), Executor 1 (EX1) and Executor 2 (EX2; Lee et al., 2007) – both required for ^1^O_2_-dependent nuclear gene expression changes and stress responses – and PRIN2, which has been shown to be part of the plastid RNA polymerase (PEP) machinery (Kindgren et al., 2012a).

A further retrograde signaling pathway appears to originate from perturbation of plastid gene expression (PGE) both at the level of transcription and translation (Sullivan and Gray, 1999; Woodson et al., 2013). *Arabidopsis* mutants defective in SIG2 and SIG6 factors have been, indeed, shown to be the source of plastid retrograde signals (Woodson et al., 2013). Moreover, based on transcriptomic analyses, the transcription-, translation- and tetrapyrrole-mediated pathways seem to converge, within the chloroplast, at the level of the GUN1 protein (Koussevitzky et al., 2007; Woodson et al., 2013). Unlike the other *GUN* genes, *GUN1* encodes a plastid-located PPR protein that is part of the transcriptionally active plastid chromosome (pTAC). However, the molecular details of GUN1 function remain elusive.

Generally speaking, the majority of tomato proteins that share homology with *Arabidopsis* retrograde signaling factors are encoded by genes that show reduced expression (with respect to leaves) in the fruits at the MG, breaker and ripening stages (**Table 2**). This is true of the tomato homologs of GUN2 (Solyc12g009470) and GUN3 (Solyc01g008930), and the subunits of the Mg-chelatase enzymes GUN4 (Solyc06g073290), GUN5 (Solyc04g015750), CHLD (Solyc04g015490), CHLI-1, and CHLI-2 (Solyc10g008740). In contrast, *GUN6* transcripts (Solyc08g065480 and Solyc08g065480) encoding ferrochelatase 1 (FC1) accumulate to relatively high levels in all fruit stages, suggesting that FC1-dependent heme synthesis might play a key role as a source of messenger molecules to coordinate plastid and nucleus activities during fruit ripening. At all events, stress-related retrograde signals like PAP and MEcPP do not appear to have a major role in fruit formation, as shown by the leaf-like levels of *HDS* transcripts (Solyc11g069380) and the increased accumulation of both SAL1 (Solyc05g056490) and XRN2 (Solyc04g049010) and XRN3 (Solyc04g081280) mRNAs in all stages of fruit differentiation and maturation. Similarly, all factors involved in redox- and ROS-mediated retrograde signals are encoded by genes that are only weakly transcribed in fruits, such as STN7 (Solyc12g021280), EX1 (Solyc01g105990), EX2 (Solyc06g071430), and PRIN2 (Solyc05g006110), further supporting the inference that stress-related pathways are not involved in the chloroplast-chromoplast transition.

Once retrograde signals have been generated, they must be exported to the nucleus and interact with transcription factors to regulate gene expression. Hence, the discovery in *Arabidopsis* of a mechanism for the transduction of a retrograde signal in the nucleus represents a major breakthrough. The GUN1-dependent retrograde pathway has recently been shown to be mediated by N-PTM, an N-terminal fragment of the transcription factor PTM that is associated with the chloroplast envelope membrane. Once formed, N-PTM is translocated to the nucleus, where it activates the expression of ABI4, an AP2-type transcription factor reported to have a general role in plastid retrograde signaling (Sun et al., 2011). This pathway, however, does not seem to play a key role during fruit maturation and ripening, as indicated by the low accumulation of Solyc10g081470 transcripts, which code for the putative homolog of PTM in tomato, at all stages of fruit development.

GLK1 and GLK2 (Golden 2-like 1 and Golden 2-like 2) are MYB-GARP transcription factors that also act downstream of plastid retrograde signaling to regulate a large set of genes encoding photosynthetic thylakoid membrane proteins (Rossini et al., 2001). Two GLK genes are found in the tomato genome (*GLK1, Solyc07g053630; GLK2, Solyc08g077230*; Powell et al., 2012), and *GLK2* accumulates during the earliest stages of fruit maturation, when new chloroplasts are needed to keep pace with cell division and expansion. Breeders have selected tomato varieties carrying light-green fruit before ripening, and Powell et al. (2012) have demonstrated recently that the light-green trait is due to the presence of a truncated version of *GLK2/Solyc08g077230*. These varieties produce fruits with a reduced sugar content, as a consequence of the reduced photosynthetic performance of the mesocarp cells. In agreement with that, overexpression of either *GLK1* and *GLK2* resulted in dark green tomato fruit with high chlorophyll and chloroplast levels in addition to more stacked thylakoid grana and elevated starch in the fruit (Nguyen et al., 2014).

Furthermore, the decrease in accumulation of *GLK2/Solyc08g-*077230 transcripts at later stages in fruit development agrees with the increased accumulation, at breaker and ripening stages, of *ABI4* (Koussevitzky et al., 2007), *HY5* and *HSP90* genes (Kindgren et al., 2012b), which are known to inhibit photosynthesis-related gene expression. This indicates that they are part of the genetic program leading to the dismantling of the thylakoid membrane and its associated photosynthetic machinery.

## CONCLUSION

In this survey we have explored the genetic and the hormonal regulation of fruit formation and development in tomato. Many players in the regulation of ripening have been identified, and their action clarified. However, the exchange of information between plastids and the nucleus has not been satisfactorily explored with regard to fruit ripening, despite the fact that the dedifferentiation of chloroplasts into chromoplasts is such a spectacular aspect of the whole process. Indeed comparative analyses reveal that several genes encoding protein involved in the retrograde and anterograde signaling undergo to transcriptional regulation and these waves can be associated to important developmental checkpoints. Indeed a better comprehension of these signaling pathways will provide new molecular tools to be used in breeding programs finalized to important applicative improvements, such as increase tomato fruit quality and tomato shelf-life.

## Conflict of Interest Statement

The authors declare that the research was conducted in the absence of any commercial or financial relationships that could be construed as a potential conflict of interest.

## Footnotes

1 http://solgenomics.net/tools/blast/

2 http://bar.utoronto.ca/efp_tomato/cgi-bin/efpWeb.cgi
